# *IL*-*17A*, *IL*-*17F* and *IL*-*23R* Gene Polymorphisms in Polish Patients with Rheumatoid Arthritis

**DOI:** 10.1007/s00005-014-0319-5

**Published:** 2014-11-12

**Authors:** Katarzyna Bogunia-Kubik, Jerzy Świerkot, Anna Malak, Barbara Wysoczańska, Beata Nowak, Katarzyna Białowąs, Katarzyna Gębura, Lucyna Korman, Piotr Wiland

**Affiliations:** 1 Laboratory of Clinical Immunogenetics and Pharmacogenetics, L. Hirszfeld Institute of Immunology and Experimental Therapy, Polish Academy of Sciences, Wrocław, Poland; 2 Department of Rheumatology and Internal Medicine, Wrocław Medical University, Wrocław, Poland; 3 Department of Pharmacology, Wrocław Medical University, Wrocław, Poland; 4 Department of Rheumatology and Internal Medicine, Wrocław University Hospital, Wrocław, Poland

**Keywords:** Th17, IL-17A, IL-17F, IL-23R, Gene polymorphism, Rheumatoid arthritis, Disease progression, Therapy with TNF-alpha inhibitors

## Abstract

Among the complex network of inflammatory cells involved in the pathogenesis of rheumatoid arthritis (RA), Th17 cells have recently been identified as key cells in the promotion of autoimmune processes, and joint destruction. The IL-23/Th17 signalling pathway, consisting of IL-23/IL-23R, IL-17A and IL-17F encoding genes, represents a candidate way for RA development with possible involvement in disease susceptibility and effect on disease progression. The present study aimed to determine the association between the polymorphic variants of the *IL*-*17A* (rs2275913), *IL*-*17F* (rs763780) and *IL*-*23R* (rs11209026) genes and RA susceptibility, progression and response to therapy with TNF-α inhibitors. Eighty-nine patients and 125 healthy individuals were investigated. The *IL*-*17A* polymorphism was found to affect RA progression and response to anti-TNF treatment. Female patients carrying the *IL*-*17A* wild-type genotype more frequently presented with stage 4 (8/24 vs. 6/47; *p* = 0.058) and were characterized by more active disease (the highest DAS28 score >5.1) after 3 months of therapy with the TNF inhibitors (12/23 vs. 15/45; *p* = 0.040). The *IL*-*17F* polymorphism appeared to be associated with susceptibility to the disease. The presence of the *IL*-*17F* minor variant (OR 3.97; *p* < 0.001) and its homozygosity (OR 29.62; *p* < 0.001) was more frequent among patients than healthy individuals. These results suggest that the polymorphisms within the *IL*-*17A* and *IL*-*17F* genes play a significant role in RA.

## Introduction

Rheumatoid arthritis (RA) is a chronic autoimmune disorder characterized by systemic inflammation and persistent synovitis that affects the joints and promotes joint destruction (Lee and Weinblatt 2001). Among the complex network of inflammatory cells involved in the pathogenesis of RA, Th17 cells have recently been identified as key cells in the promotion of autoimmune processes, joint destruction and angiogenesis (Annunziato et al. 2009). Th17 cells and their cytokines are associated with several autoimmune and inflammatory diseases, such as RA, systemic lupus erythematosus, multiple sclerosis (MS), psoriasis, inflammatory bowel disease (IBD) and allergy and asthma (Miossec et al. 2009; Wilke et al. 2011). The hallmark of the Th17 subset is the production of interleukin IL-17A and IL-17F, which share strong homology, and surface expression of the IL-23 receptor (IL-23R) (Hot et al. 2011). Therefore, IL-17A, IL-17F and IL-23 may play an important role in T cell-triggered inflammation by upregulating some of the gene products involved in cell activation, proliferation and growth, and it is an important inductor of various cytokines and chemokines that are crucial in regulating the inflammatory response. IL-23 plays a key role in the development of pathogenic Th17 cells that produce the cytokine IL-17, which induces the production of several pro-inflammatory cytokines, such as TNF-α and IL-6, chemokines, and some other additional novel factors responsible for RA and other autoimmune diseases (Bettelli et al. 2008; Langrish et al. 2005). IL-23 is an essential promoter of chronic joint inflammation and mediates proinflammatory activity, in part, via production of IL-17 through Th17 lymphocyte activation (Aggarwal et al. 2003; McKenzie et al. 2006). Interestingly, in RA, IL-23 levels correlate with IL-17 levels in the joint fluid and with IL-17 and TNF-α levels in the serum (Wendling 2008). In addition, the serum level of IL-23 in patients with RA is correlated with the number of swollen joints, the Disease Activity Scores of 28 (DAS28) joints, and the serum level of IL-17. IL-17 has also been found in the synovial membrane and synovial fluid of RA patients (Kotake et al. 1999; Li et al. 2013).

Thus, the IL-23/Th17 signalling pathway, consisting of IL-23/IL-23R, IL-17A and IL-17F encoding genes, represents a candidate way for RA development with possible involvement in disease susceptibility and effect on disease progression (Iwakura and Ishigame 2006; Lubberts 2010).

Thus in the present study the associations between genetic variants within the IL-23/Th17 signalling pathway were analysed. We aimed to assess the significance of three biallelic polymorphisms: *IL*-*17A* (rs2275913; G-197A), *IL*-*17F* (rs763780; A7488G; His161Arg) and *IL*-*23R* (rs11209026, G1142A; Arg381Gln) for RA susceptibility, progression of the disease and response to therapy with TNF-α inhibitors.

## Materials and Methods

### Patients and Controls

For the study 89 patients (female/male: 72/17) diagnosed with RA and hospitalized at the Rheumatology Clinic of the Medical University in Wroclaw, Poland were included. The following inclusion criteria were accepted: consent to participate in the study; confirmed RA based on criteria of the American College of Rheumatology; active form of the disease: DAS28 > 5.1; age over 18 years; women and men with reproductive potential had to use reliable contraception; taking nonsteroidal anti-inflammatory drugs and glucocorticosteroids in stable doses was allowed.

There were the following exclusion criteria: pregnancy or breastfeeding; coexistence of other systemic diseases of connective tissue besides RA; clinically significant impairment of hepatic and renal function; alcohol abuse; infection with hepatotropic viruses; infections resistant to therapy; ongoing history of cancer if no cure was achieved; uncontrolled diabetes; patient unwilling or unable to cooperate.

Patients who had been treated with recommended doses of TNF-α inhibitors (adalimumab, etanercept, infliximab, certolizumab) for at least 3 months or had stopped therapy because of adverse events were investigated. To examine the response to anti-TNF therapy in RA, blood samples, laboratory data and clinical data were collected at baseline (prior to anti-TNF therapy) and 3 months after treatment. Clinical evaluation was based on medical history, number of painful and swollen joints, pain intensity assessed by the patient on a 100-mm visual analogue scale and laboratory tests (ESR, CRP). The parameters allowed determination of improvement according to the criteria based on DAS28 suggested by the European League Against Rheumatism.

All the patients provided written informed consent. The study was approved by the Wroclaw Medical University Ethics Committee. For patient characteristics see Table 1.

**Table 1:** Characteristics of RA patients

Variable	RA patients (*N* = 89)
Sex (female/male); *n* (%)	72 (81 %)/17 (19 %)
Age at RA onset, mean (range) years	38 (15–65)
Disease duration, mean (range) years	13 (1–39)
Rhemathoid factor positive^a^, *n* (%)	73 (91 %)
Anti-CCP present^b^, *n* (%)	49 (89 %)
Stage, *n* (%)	
1	1 (1.1 %)
2	21 (23.6 %)
3	53 (59.6 %)
4	14 (15.7 %)
DAS28 after 3 months of anti-TNF treatment^c^, *n* (%)	
≤2.6	2 (2.4 %)
2.6 < DAS28 ≤ 3.2	2 (2.4 %)
3.2 < DAS28 ≤ 5.1	42 (50 %)
>5.1	38 (45.2 %)

^a^Data available for 80 patients

^b^Data available for 55 patients

^c^Data available for 84 patients

Stages of RA were assessed according to Wheeless (2012). According to this classification the first stage RA is characterized by synovitis, or an inflammation of the synovial membrane, causing swelling of involved joints and pain upon motion. However, there is no x-ray evidence of joint destruction, with the exception of swelling of soft tissues or early stages of osteoporosis. In stage II, there is a spread of inflammation in synovial tissue, affecting joint cavity space across joint cartilage. This inflammation will gradually result in a destruction of cartilage, accompanied by a narrowing of the joint. Severe RA, stage III, is marked by formation of pannus in the synovium. Loss of joint cartilage exposes bone beneath the cartilage. These changes will become evident on x-ray, along with erosions and signs of deformation. Stage IV is called terminal or end stage RA. The inflammatory process has subsided and formation of fibrous tissue and/or fusing of bone results in ceased joint function. Rheumatoid nodules may also be present in patients in stage IV of the disease.

In addition 125 Polish healthy individuals of both sexes (female/male: 63/62) served as controls.

### *IL*-*17A*, *IL*-*17F* and *IL*-*23R* Genotyping

Three biallelic polymorphisms were studied: *IL*-*17A* (rs2275913; G-197A), *IL*-*17F* (rs763780; A7488G; His161Arg) and *IL*-*23R* (rs11209026, G1142A) as previously described (Wróbel et al. 2014). In brief, DNA was extracted from peripheral blood taken on EDTA using the Maxwell 16 Blood DNA Purification Kit (Promega Corp., Madison, WI, USA) following the recommendations of the manufacturer.

The *IL*-*17F* (rs763780; A7488G) polymorphism was analysed using a polymerase chain reaction (PCR) restriction fragment length polymorphism assay, which amplified a fragment of the promoter region of the gene using primers as previously described (Saitoh et al. 2011) (forward: 5′-GTT CCC ATC CAG CAA GAG AC-3′, and reverse: 5′-AGC TGG GAA TGC AAA CAA AC-3′). The PCR conditions were as follows: 94 °C for 3 min; 35 cycles at 94 °C for 30 s, 60 °C for 30 s and 72 °C for 30 s; and a final elongation step at 72 °C for 7 min. The PCR products were digested with the *Nla*III restriction endonuclease (New England BioLabs Inc.) and analysed in 2 % agarose gel stained with ethidium bromide and visualized under UV light. Three patterns were observed following digestion and electrophoresis: a single 412 bp fragment (individuals homozygous for the *IL*-*17F G* allele, lacking the *Nla*III site), three fragments of 412, 288 and 124 bp in length (heterozygous individuals) or two fragments of 288 and 124 bp (individuals homozygous for the *IL*-*17F A* allele).

PCR amplifications for the *IL*-*17F* gene polymorphism studies were carried out in the 2,720 Thermal Cycler (Applied Biosystems, Foster City, USA).

The *IL*-*17A* (rs2275913; G-197A) and *IL*-*23R* (rs11209026, G1142A) alleles were determined by real-time PCR amplifications and analysis of the typing results were performed using Roche LightCycler 480 instrument. The LightSNiP (rs2275913) assay designed by TIB MOLBIOL (GmbH, Berlin, Germany) or TaqMan SNP Genotyping Assay (rs11209026) (Life Technologies, USA) was used for detection of *IL*-*17A* and *IL*-*23R* alleles, respectively.

The *IL*-*17F* and *IL*-*23R* polymorphisms were analysed in 89 patients and 125 healthy individuals while the *IL*-*17A* genotyping was performed for 88 RA patients (71 women and 17 men) and 125 controls.

### Statistical Analysis

Genotype and allele frequencies were compared between the study groups by the *χ* ^2^ test with Yates correction or Fisher’s exact test when necessary using Statistica 5.5 for Windows software. The odds ratio (OR) was calculated by Haldane’s modification of Woolf’s method and the significance of its deviation from unity was estimated by Fisher’s exact test. All *p* values were corrected for the number of comparisons. Probability values <0.05 were considered statistically significant.

## Results

### Distribution of *IL*-*17A* and *IL*-*23R* Alleles and Genotypes in Patients and Controls

*IL*-*17A* and *IL*-*23R* alleles and genotypes segregated similarly in patients and controls.

The *IL*-*17A* (rs2275913) *GG*, *GA* and *AA* genotypes were detected in 12 (13.6 %), 44 (50 %) and 32 (36.4 %) patients, and in 20 (16 %), 67 (53.6 %) and 38 (30.4 %) controls, respectively (Table 2). The allelic frequencies of the *A* variant of the *IL*-*17A* gene were 0.614 and 0.572, in patients and controls, respectively, which closely resemble those observed in other studies of the healthy European populations.

**Table 2:** Distribution of the *IL*-*17A*, *IL*-*17F* and *IL*-*23R* alleles and genotypes in Polish patients with RA and healthy individuals

Polymorphism	RA patients		Controls		OR, *p*
	*N*	%	*N*	%	
*IL*-*17A* (G-197A) rs2275913					
*GG*	12	13.6	20	16.0	
*GA*	44	50.0	67	53.6	
*AA*	32	36.4	38	30.4	
*G*	56	63.6	87	69.6	
*A*	76	86.4	105	84.0	
*IL*-*17F* (A7488G) rs763780					
*AA*	64	71.9	114	91.2	
*AG*	16	18.0	11	8.8	
*GG*	9	10.1	0	0	OR 29.62, *p* = 0.0003, *p*_*c*_ = 0.0009
*A*	80	89.9	125	100	
*G*	25	28.1	11	8.8	OR 3.97, *p* = 0.0002, *p*_*c*_ = 0.0006
*IL*-*23R* (G1142A) rs11209026					
*GG*	83	93.3	111	88.8	
*GA*	6	6.7	14	11.2	
*AA*	0	0	0	0	
*G*	89	100	125	100	
*A*	6	6.7	14	11.2	

*p*_*c*_−*p* values corrected for the number of comparisons

The *IL*-*23R A* variant was very rarely detected. None of the individuals tested was carrying the *IL*-*23R* *AA* homozygous genotype. Eighty-three patients (93.3 %) and 111 (88.8 %) healthy individuals were homozygous for the *IL*-*23R* (rs11209026) *G* wild-type allele. There were only 6 (6.7 %) and 14 (11.2 %) *GA* heterozygotes among RA patients and controls, respectively (Table 2). The allelic frequencies of the *A* variant of *IL*-*23R* were 0.034 and 0.044, in patients and controls, respectively.

### Associations with Predisposition to the Disease, Impact of the *IL*-*17F* Polymorphism

As expected, the occurrence of RA was more frequent among female than male patients as compared to controls (72/89 vs. 63/125, OR 4.08; *p* < 0.001).

Among the polymorphisms studied, *IL*-*17F* (A7488G) polymorphism was found to be associated with RA. The presence of the *IL*-*17F G* variant was more frequently observed among patients than healthy individuals. This allelic variant was detected in 25 out of 89 patients with RA and only in 11 out of 125 controls (OR 3.97; *p* < 0.001). The allelic frequencies of the *G* variant of the *IL*-*17F* gene were 0.191 and 0.044, in patients and controls, respectively.

An even stronger association was observed when the *IL*-*17F GG* homozygous genotype was considered. The *IL*-*17F GG* homozygosity was detected in 9 RA patients (7 females and 2 men) and none of the controls (OR 29.62; *p* < 0.001) (Table 2). Similar relationships were observed when patients and controls were stratified with respect to sex (OR 13.20, *p* = 0.021 and OR 14.54, *p* = 0.071, for females and men, respectively).

### Relationships of the *IL*-*17A* Genotypes with Disease Progression and Anti-TNF Treatment

There were only females among RA patients with stage 4 of the disease (14/72 vs. 0/17, for stage 4 cases among female and male patients; *p* = 0.063).

**Fig. 1:**
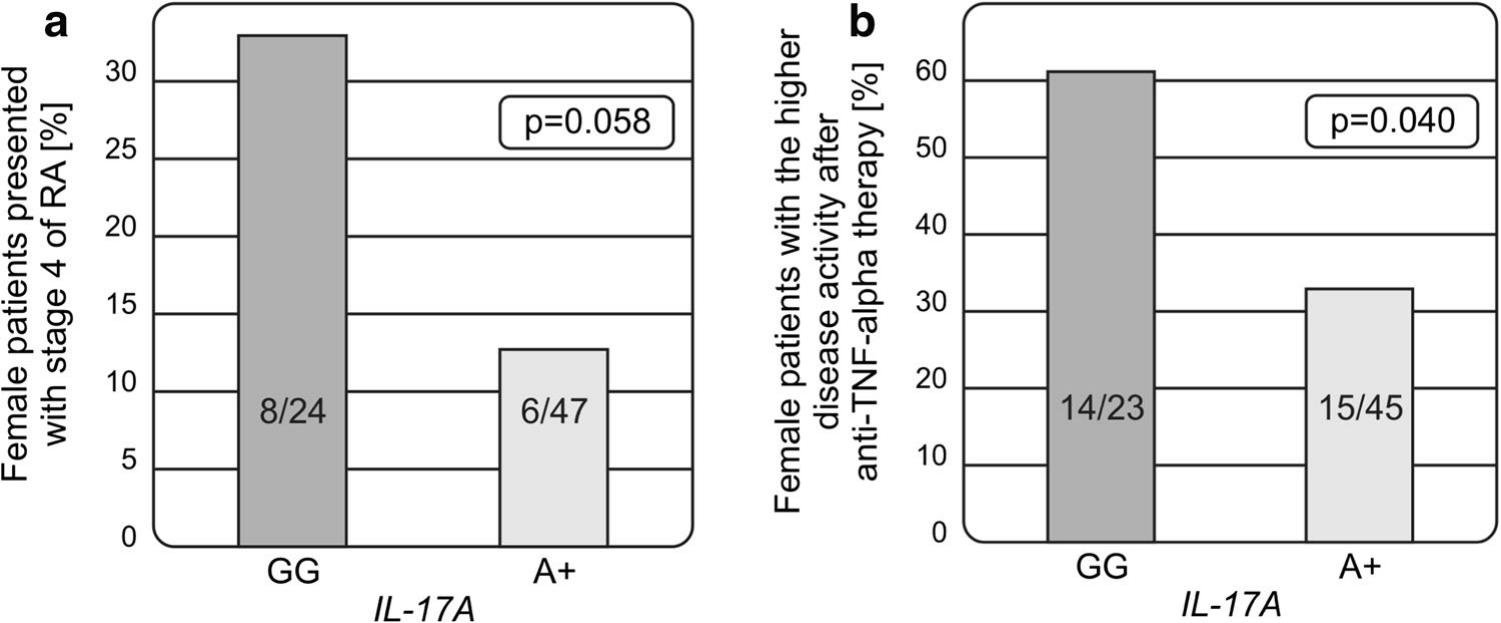
The associations of the *IL*-*17A* (rs2275913; G-197A) polymorphism with disease progression (**a**) and therapy with TNF-α inhibitors (**b**) in women with RA. The *IL*-*17 GG* wild-type genotype was more frequently detected among female patients with the most advanced RA and the highest activity of the disease after 3 months of anti-TNF treatment

Interestingly, the majority of these women were carrying the *IL*-*17A* *GG* wild-type homozygous genotype. Among female patients, 8 out of 24 with the *IL*-*17A GG* genotype and 6 out of 47 carrying the *A* variant (*GA* or *AA* genotypes) were in the most advanced stages of the disease (8/24 vs. 6/47, *p* = 0.058; Fig. 1a).

Moreover, female patients with the *IL*-*17 GG* wild-type genotype had more active disease (the highest DAS28 activity score > 5.1) after 3 months of therapy with TNF inhibitors. Among 23 female patients carrying the *IL*-*17 GG* genotype, 14 had the most active disease as compared to 15 out of 45 women with the *IL*-*17 A* variant (14/23 vs. 15/45, *p* = 0.040; Fig. 1b).

### Relationships with the *IL*-*23R* Polymorphism

The presence of the minor allele of the *IL*-*23R* polymorphism was slightly more frequently observed among female patients in stage 3 or 4 of the disease. All women in stage 3 or 4 were carrying the *IL*-*23R G* allele (were homozygotes) as compared to 49 out of 67 women (70 %) with wild-type genotype (*p* = 0.164). Of note, patients homozygous for the major allele were reported to have significantly higher serum IL-17A concentrations compared with patients with the minor allele (Hazlett et al. 2012). Thus lower IL-17A producers seemed to prevail among women in stage 3 or 4 of the disease.

As the presence of the *IL*-*17A* -*197A* allele in healthy individuals was also found to be associated with increased IL-17A production (Espinoza et al. 2011), in further analyses we considered both genotypes associated with increased IL-17A expression (homozygosity for the major rs11209026 *IL*-*23R* allele, the presence of the minor rs2275913 *IL*-*17A* allele).

Female patients lacking both high-producer genotypes were more frequently observed among women in stage 4 disease (37/43 vs. 20/28; *p* = 0.220) and those with more advanced disease (with DAS28 > 5.1; 13/28 vs. 25/40; *p* = 0.220). However, these relationships did not reach statistical significance. Thus it seemed that the presence of the *IL*-*23R* high-producer genotype in patients carrying the *IL*-*17A* wild-type genotype weakened the (described in the previous paragraph) association with the *IL*-*17A* polymorphism.

## Discussion

The polymorphisms located within genes coding for IL-17A and IL-17F appeared to play a role as genetic factors associated with susceptibility to RA, disease progression and response to treatment in the Polish population.

In the present study, the rs763780 *IL*-*17F* (A7488G) polymorphism was found to be associated with susceptibility to RA, and the presence of the *G* variant (and the *GG* genotype) was significantly more frequently observed in patients than in healthy individuals. This is a novel observation not previously described.

The association of the *IL*-*17F* (A7488G) polymorphism with susceptibility to the disease was reported, e.g. for the development of asthma (Kawaguchi et al. 2006; Qian et al. 2012), IBD (Arisawa et al. 2008; Seiderer et al. 2008), autoimmune thyroid diseases (Yan et al. 2012) and the most recently with MS (Wang et al. 2014) and acute myeloid leukaemia (Wróbel et al. 2014).

As for the RA patients, this polymorphism was also studied by Paradowska-Gorycka et al. (2010b) together with another coding substitution within the third exon of the *IL*-*17F* gene (rs2397084; A7383G; Glu26Gly). The authors did not find any association with susceptibility to the disease. However, their detailed genotype–phenotype analysis indicated that the *IL*-*17F G* (161Arg) variant of the *IL*-*17F* (rs763780) polymorphism might be associated with disease activity. Its presence was found to be correlated with a higher number of tender joints, higher mean value of DAS28-CRP and higher health assessment questionnaire score (Paradowska-Gorycka et al. 2010b).

Interestingly, Kawaguchi et al. (2006) showed the functional consequences of this *IL*-*17F* polymorphism and suggested that the IL-17 expression and activity may be suppressed in carriers of the rare *G* allele.

Taking the evidence together, the presence of the rare *IL*-*17F G* variant (rs763780) associated with lower expression of IL-17F was found to affect the disease susceptibility (present study) and activity of RA (Paradowska-Gorycka et al. 2010b) in the Polish population.

In our study we also found that the rs2275913 *IL*-*17A* (G-197A) polymorphism was associated with disease progression and the response to therapy with TNF-α inhibitors. These associations were observed especially in female patients carrying the wild-type homozygous genotype who more frequently presented with the most aggressive/advanced disease and characterized by a poor response to the anti-TNF treatment. These relationships have not been described previously in the literature.

So far, only three studies, by Furuya et al. (2007), Nordang et al. (2009) and the present one, have considered the role of the *IL*-*17A* promoter polymorphism in RA. The previous studies showed a weak but significant correlation with the rs2275913 *IL*-*17A* promoter polymorphism in Norwegian patients with RA (Nordang et al. 2009) and the association of the rs3804513 *IL*-*17A* (A52053197T, A > T) gene polymorphism with radiographic progression in Japanese patients with early RA (Furuya et al. 2007).

As for the relationships with susceptibility or progression of other autoimmune diseases the associations of the *IL*-*17A* rs2275913 variants with ulcerative colitis in Koreans (Kim et al. 2011) and Japanese (Hayashi et al. 2013), but not for our Caucasian population, were described. In the latter study *IL*-*17A* polymorphism was also found to be associated with the noncontinuous and pancolitis phenotypes of ulcerative colitis (Hayashi et al. 2013). Interestingly, this polymorphism was also described to affect carcinogenesis. For example, some associations with the risk of gastric cancer, the development of intestinal-type cancer or diffuse-type cancer, and gastric mucosal atrophy were reported (Shibata et al. 2009).

We observed a higher frequency of females carrying the wild-type genotype of the rs2275913 *IL*-*17A* polymorphism among patients with the highest stages of the disease and in those with the most active disease after 3 months of anti-TNF-α treatment. This is a novel observation not previously described.

Interestingly, Espinoza et al. (2011) demonstrated that in vitro stimulated T cells from healthy individuals possessing the *197A* allele produced significantly more IL-17 than those without the *197A* allele. Thus, in our study the potential low producers of IL-17A appeared to exhibit worse prognosis. It is difficult to discuss this relationship. First of all, it would be of interest to assess whether the association between *IL*-*17A* gene polymorphism and expression observed in healthy individuals (Espinoza et al. 2011) is also valid in RA patients, and furthermore to verify the association after subsequent 3 and 6 months of anti-TNF treatment. These issues warrant further studies. Moreover, it should be taken into account that in RA IL-17A acts as an important component of a more complex cytokine network involving such factors as proinflammatory cytokines (TNF-α and IL-6) or chemokines (Bettelli et al. 2008; Langrish et al. 2005). Thus the observed relationship could be more complex. Nevertheless, our results imply that polymorphism located within the IL-17A encoding gene may have prognostic value for patients with RA.

We did not find any significant association with the rs11209026 *IL*-*23R* polymorphism with RA. Our observation concurs with the other studies. The results of very recent meta-analyses suggested that other polymorphisms located within the *IL*-*23R* gene were potentially associated with the development of RA in Europeans, including the *IL*-*23R* rs134315, rs10489629 and rs7517847 polymorphisms (Song et al. 2012; Zhai et al. 2012).

There are, however, a few reports that showed the correlation of the rs11209026 *IL*-*23R* polymorphism with the development and course of other human autoimmune disorders, such as psoriatic arthritis (Hinks et al. 2011) or ankylosing spondylitis (Sáfrány et al. 2009).

Moreover, the rs11209026 *IL*-*23R* polymorphism was reported to affect the IL-17A serum level in RA patients. Patients homozygous for the major allele had significantly higher serum IL-17A concentrations compared with patients with the minor allele (Hazlett et al. 2012). Therefore, in our study we also considered the genotypes associated with increased IL-17A expression (homozygosity for the major rs11209026 *IL*-*23R* allele and the presence of the minor rs2275913 *IL*-*17A* allele). However, no significant relationships were detected.

In summary, we were able to detect some associations with *IL*-*17A* and *IL*-*17F* but not *IL*-*23R* gene polymorphism in Polish patients with RA. These relationships might be attributed to the fact that epidemiological results often do not coincide with functional studies because RA is a complex disease with contributions from multiple genes, different genetic backgrounds and environmental factors. One of the factors that could be considered is the signal transducer and activator of transcription (STAT)3 belonging to STAT proteins that play a key role in mediating signals during Th1, Th2 and Th17 differentiation. STAT3 has critical functions in development of IL-17-secreting Th cells in the cytokine environment or the expression of IL-17A, IL-17F, and retinoic acid-related orphan receptor (ROR-γt) in Th17 cultures (Paradowska-Gorycka et al. 2010a, b; Yang et al. 2007). According to our knowledge, these relationships have not been analysed so far.

Obviously, these results should be confirmed in a more extended study, including patients from other centers. A relatively small size of patients cohort analysed in the present study constitutes the most important limitation of our work.

It would also be of interest to relate the results of the polymorphism studies with the expression and serum concentration of IL-17A, IL-17F and IL-23, and other proinflammatory cytokines, such as TNF-α.

In conclusion, the results of our study strongly suggest that the polymorphisms within the *IL*-*17A* and *IL*-*17F* genes affect the susceptibility to RA, disease progression and response to treatment with anti-TNF inhibitors.
